# The environmental controls on efficiency of enhanced rock weathering in soils

**DOI:** 10.1038/s41598-023-36113-4

**Published:** 2023-06-16

**Authors:** Hang Deng, Eric Sonnenthal, Bhavna Arora, Hanna Breunig, Eoin Brodie, Markus Kleber, Nicolas Spycher, Peter Nico

**Affiliations:** 1 Department of Energy and Resources Engineering, College of Engineering, Peking University, Beijing, 100871 China; 2 Earth and Environmental Sciences Area, Lawrence Berkeley National Laboratory, Berkeley, CA 94720 USA; 3 Energy Analysis and Environmental Impacts Division, Energy Technology Area, Lawrence Berkeley National Laboratory, Berkeley, CA 94720 USA; 4 Department of Crop and Soil Science, College of Agricultural Sciences, Oregon State University, Corvallis, OR 97331 USA

**Keywords:** Biogeochemistry, Environmental sciences, Solid Earth sciences, Engineering

## Abstract

Enhanced rock weathering (ERW) in soils is a promising carbon removal technology, but the realistically achievable efficiency, controlled primarily by in situ weathering rates of the applied rocks, is highly uncertain. Here we explored the impacts of coupled biogeochemical and transport processes and a set of primary environmental and operational controls, using forsterite as a proxy mineral in soils and a multiphase multi-component reactive transport model considering microbe-mediated reactions. For a onetime forsterite application of ~ 16 kg/m^2^, complete weathering within five years can be achieved, giving an equivalent carbon removal rate of ~ 2.3 kgCO_2_/m^2^/yr. However, the rate is highly variable based on site-specific conditions. We showed that the in situ weathering rate can be enhanced by conditions and operations that maintain high CO_2_ availability via effective transport of atmospheric CO_2_ (e.g. in well-drained soils) and/or sufficient biogenic CO_2_ supply (e.g. stimulated plant–microbe processes). Our results further highlight that the effect of increasing surface area on weathering rate can be significant—so that the energy penalty of reducing the grain size may be justified—only when CO_2_ supply is nonlimiting. Therefore, for ERW practices to be effective, siting and engineering design (e.g. optimal grain size) need to be co-optimized.

## Introduction

In order to achieve stringent climate goals, such as limiting global warming to 1.5 °$\mathrm{C}$, hundreds of gigatonnes (Gt) of CO_2_ need to be captured and stored^1,2^. To achieve this objective, a wide range of carbon dioxide removal (CDR) technologies would have to be developed and deployed at scale^2,3^. Among them, enhanced rock weathering (ERW) is a competitive option because it combines direct removal of atmospheric CO_2_ with long-term storage through conversion into aqueous alkalinity or carbonate minerals^3–14^. Theoretical estimates have shown that the weathering of 1 tonne of basic (e.g. basalts) and ultrabasic rocks (e.g. olivine) can remove ~ 0.3 and 0.8 tonne of CO_2_, respectively^8^, indicating a considerable overall theoretical potential^4,8,15^. ERW can also mitigate ocean acidification^10^, and has an intermediate cost compared to other CDR technologies^3,12^.

ERW in soils has a series of additional benefits^16^. It takes advantage of soil chemistry, e.g. elevated local CO_2_ concentration and the presence of organic ligands from plant and microbial activities, to accelerate the weathering reactions, while not competing with other land uses^7,9,17^. Application of ground rocks to ~ 50% of global croplands would potentially sequester up to 2 Gt of CO_2_ per year^5^. ERW in soils can improve soil quality by reducing soil acidity, which has been traditionally achieved by the practice of liming^18,19^, while avoiding CO_2_ emissions associated with lime application^6^. ERW can also release macro and micro nutrients and thereby reduce the need of soil fertilization with fertilizers, thus improving productivity across agroecosystems^12,20–22^.

However, for ERW to be deployed at a meaningful scale, the primary challenge is to achieve and maintain high weathering rates and thus carbon removal efficiency. In current analyses, weathering rates have been identified as a major source of uncertainty^10,12^. In situ weathering rates in the soil environment are particularly variable owing to the complex nature of the biogeochemical reactions in the soil matrix and their interactions with the applied rock materials^15,23^.

Theoretical weathering rates that are based on batch experiments or reaction rate laws can be helpful^14,24,25^, but one of the caveats is that they do not account for factors such as transport limitation. Transport limitations to reactants and reaction products are a major constraint on achievable reaction rates, resulting in observed weathering rates that are orders of magnitude lower than the theoretical estimates^19,26^. This discrepancy is particularly relevant for the optimization of particle size of rock materials for field application given that the energy penalty of grinding and comminution to create more surface area is significant^27^. The complex feedback between characteristics of rock materials, soil matrix, and transport is further emphasized by the observation that experimental weathering rates may increase when application rates decline, as shown for olivine^6^. Accordingly, the overarching goal of this work is to establish (i) a mechanistic understanding as well as (ii) quantitative approximations of the extent to which both, biogeochemical reactions and transport, are affected by different environmental controls.

While existing dedicated experiments on enhanced rock weathering in soils are few, and more (laboratory and field) experiments are needed to uncover the mechanisms underlying in situ weathering rates, reactive transport modeling provides a valuable tool to complement experimental studies^5,28^. It has the advantages of expanding the spatial scales and extending beyond the duration of current experimental investigations which are typically carried out on monthly time scales, and thus to better inform field studies and engineering practices. It can also help assess potential environmental risks that can arise from the weathering products (e.g. heavy metal)^7,11^, and quantify the amount of CO_2_ that is captured and stored, because reactive transport modeling can readily track the fate of different chemical species in the system. However, modeling studies to date rarely consider reaction networks that reflect the complex soil chemistry.

To explore the major controlling chemical-physical factors of weathering rates in the soil environment, we used a 1D reactive transport model that considers a reaction network capable of capturing microbe-mediated reactions that control the soil chemistry^29,30^, and different unsaturated hydrological scenarios of the soil environment. There are numerous possibilities regarding the choice of mineralogy for EWR applications. Given our interest in probing the potentials of the ERW practice, we elected to evaluate the fate of a mineral phase that can be considered as particularly effective in producing alkalinity/carbonate rocks: the olivine group mineral forsterite. To constrain model complexity and to increase our ability to detect fundamental relationships, we limited the number of secondary solid phases, aware that this choice constitutes a likely simplification of outcomes in the natural weathering environment. As for system context, our work leverages information (e.g. composition and chemistry) for a silt loam and a sandy loam from a previous study on groundwater recharge in the Central Valley area of California^30^. These sites also convey practical relevance to our study given the interests of California in carbon neutrality^31^ and the need to employ different soil types for meaningful carbon removal via ERW.

## Methods

### Model description

The multi-component multiphase reactive transport code TOUGHREACT^32^ was used to simulate a 2-m long vertical 1D soil column under a range of conditions. It was assumed that there were no gas pressure or temperature gradients, which was done by solving Richards’ equation (i.e. module EOS9 of TOUGHREACT^33^). Diffusive transport of the gas phase was considered, as the partial pressure of CO_2_ in biologically active soil is different from that in the atmosphere. Kinetic mineral reactions were calculated using the Transition State Theory rate law^24^. The rates of microbe-mediated reactions that are important in the soil environment were tracked by a general rate law based on the Monod equation. Cation exchange was also included following the Gaines-Thomas convention. Details of the model are documented in the SI.

The computational domain was discretized into 36 grid cells. The first grid cell at the top close to the atmosphere is 2 mm, and the cell size increases gradually by ~ 20% consecutively towards the bottom, reaching 100 mm in grid cell 22. The size of grid cell 23–36 was set at 100 mm. At the top of the computational domain, the gas pressure was set equal to the atmospheric pressure. A constant water flux was introduced at the top to simulate infiltration. Water-table conditions were imposed at the bottom by setting the water saturation to one and the total pressure to atmospheric pressure.

### Simulation setup

Simulations that mimic soil column experiments with and without the application of silicate rock materials were performed, to evaluate the weathering rate of the applied rock materials and the resulting changes in soil chemistry. The applied material is forsterite (Mg_2_SiO_4_), an end member of the olivine group minerals.

The silt loam and the sandy loam, represent a less well-drained and a well-drained soil (see hydraulic properties in Table S1), respectively. Two infiltration rates (200 and 2000 mm/yr) were considered to bracket common scenarios^5,34^. Together, four water content profiles were generated (Fig. S1).

The two soils are composed of primarily quartz, some montmorillonite, and small fractions of K-feldspar, ferrihydrite and calcite (Table S2). Five secondary minerals, amorphous silica, illite, kaolinite, magnesite and siderite were included. The first three are common products of rock weathering, and magnesite and siderite are potential carbon sinks given the presence of magnesium and iron. Magnesite is the most stable magnesium carbonate but forms slowly under ambient conditions. But the carbonation rate can be significantly enhanced by organic additives and microbes^35,36^, as can be encountered in the soil environment. Therefore, magnesite was included here instead of other faster forming but metastable MgCO_3_ to represent a scenario in which carbonation is enhanced. The kinetic coefficients for all the mineral reactions are summarized in Table S3^37^.

In our simulations, the soil has an equivalent specific surface area of 10 m^2^/g^38^, and was divided among the primary minerals and secondary minerals based on their respective volume fraction. The total exchange site density was set to 10 meq/100 g soil for both soil types^39^. Chemical compositions of the initial and infiltrating water are detailed in Table S5.

The solid organic carbon pool in the soil was represented by cellulose. It was modeled to degrade into acetate following the reaction:

1 $$C{H}_{2}O\left(s\right)\leftrightarrow 0.5C{H}_{3}CO{O}^{-}+0.5{H}^{+}.$$

This reaction was maintained at equilibrium to provide a constant source of acetate. A network of kinetic reactions (Table S4) was used to simulate the degradation of acetate and thus, indirectly, the kinetic decomposition of cellulose and other important microbe-mediated reactions in the soil. This representation of soil chemistry was initially developed and tested for a site at Rifle Colorado^29^, and later adopted in the study of California Central Valley^30^. In our study, the kinetic coefficient for the microbial oxidation of acetate by oxygen (${k}_{ox}$), which is the primary reaction (Eq. (2)) that controls the soil CO_2_ profile, was re-calibrated for our hydrological scenarios to capture key features of CO_2_-depth profiles in typical field observations^40^.

2 $$C{H}_{3}CO{O}^{-}+2{O}_{2}\to 2HC{O}_{3}^{-}+{H}^{+}.$$

We considered a onetime application of 160 tonnes forsterite per hectare (i.e. ~ 16 kg/m^2^), which is equivalent to a surficial application of 1 cm of rock materials with a porosity of 50%. This application rate is also comparable to previous numerical and experimental studies^5,6,17,41^, and is within the range that would be needed if olivine weathering were to be leveraged for addressing phosphorous deficiency^21^. Two additional application depths (15 and 50 cm) were simulated, with the same total forsterite amount of 160 tonnes per hectare. In these two cases, the applied rock was mixed with the soil matrix uniformly within the application layer. It was assumed that the soil hydraulic properties were not affected by the mixing.

For the four combinations of hydraulic conditions, three rock application scenarios and a baseline case without rock amendments were simulated. For all cases with rock amendments, three forsterite surface area values (0.1, 1, and 10 m^2^/g, corresponding to grain sizes from several hundreds of microns to microns^5,42,43^) were compared. For simulations with an application depth of 15 cm and a specific surface area of 10 m^2^/g for forsterite, two elevated ${k}_{ox}$ values (10× and 100×) were also tested. For a subset of the cases (i.e. forsterite surface area = 10 m^2^/g), magnesite precipitation was suppressed by reducing its default surface area of 10 m^2^/g by 1000 times. Simulations were run to cover a total period of 5 years, which has been used as a reasonable timeframe for comparing weathering rates and analyzing carbon removal efficiency^19^.

## Results and discussion

### Changes in soil chemistry from forsterite weathering

**Figure 1:**
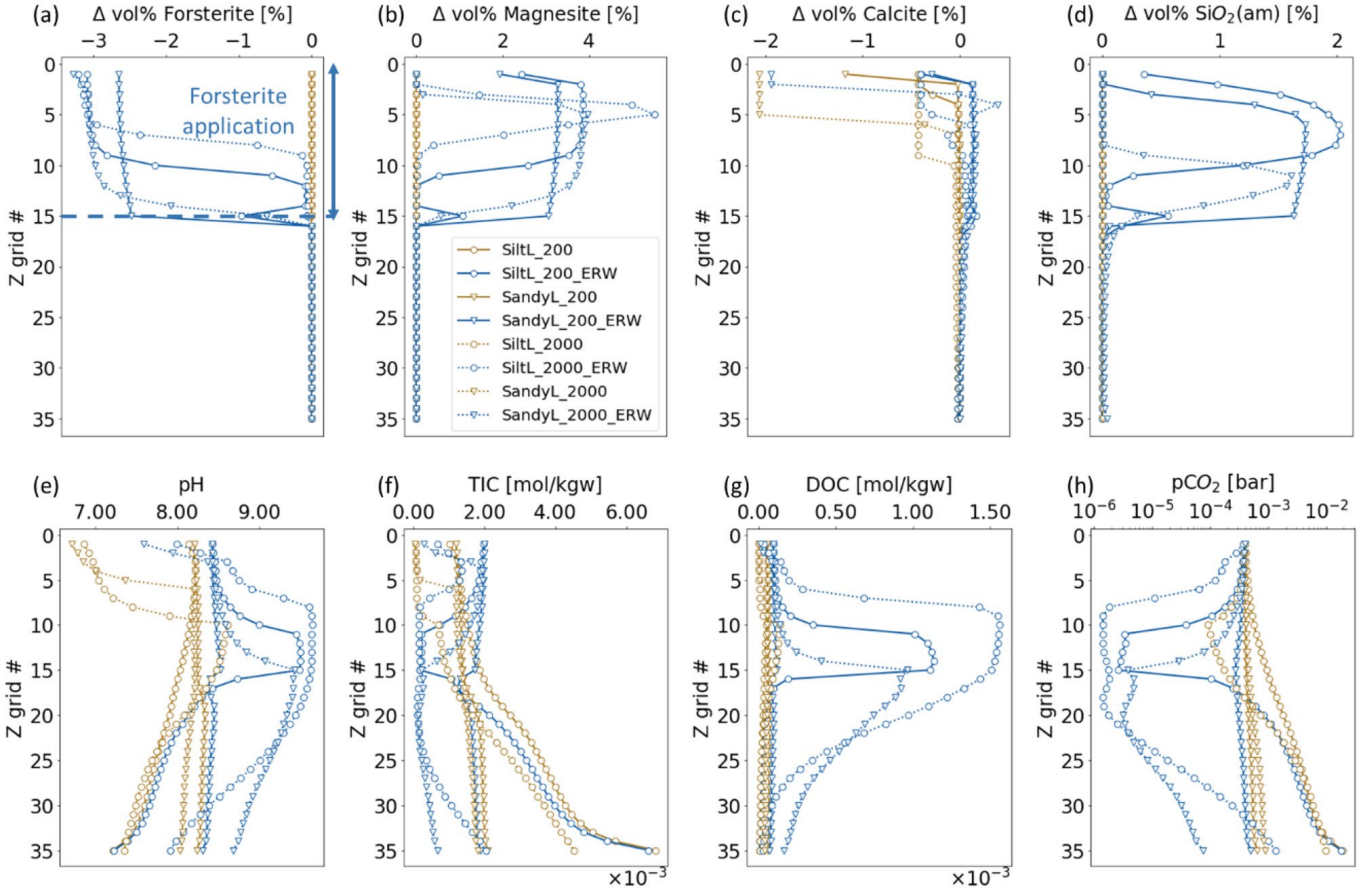
Vertical profiles at year five for an application of forsterite with a specific surface area of 10 m^2^/g in the top 15 cm for both soil types at both infiltration rates. (**a**–**d**) Changes in the volume fractions of key minerals (from left to right: forsterite, magnesite, calcite, amorphous silica), and (**e**–**h**) soil water chemistry (from left to right: pH, alkalinity as Total Inorganic Carbon (TIC), DOC as acetate) and CO_2_ partial pressure [bar].

With the application of forsterite, evident changes were observed in both the mineral phases and the aqueous composition in all cases, compared to the corresponding baseline simulations without forsterite application (Fig. 1). The major driver is forsterite dissolution, which resulted in elevated pH, and silica and Mg concentration in solution:

3 $$M{g}_{2}Si{O}_{4}\left(s\right)+4{H}^{+}\leftrightarrow Si{O}_{2}\left(aq\right)+2M{g}^{2+}+2{H}_{2}O.$$

This reaction drives the precipitation of magnesite (MgCO_3_) and amorphous silica (SiO_2_(am)) (Fig. 1b,d), which were not observed in the baseline simulations. Given the large surface areas and fast reaction rates used, the precipitation occurred primarily in the region where Mg^2+^ and SiO_2_(aq) were produced by forsterite dissolution. For calcite, montmorillonite and k-feldspar, both dissolution and precipitation were observed, whereas in the baseline simulations precipitation was absent or in smaller amounts (Fig. S2). Precipitation of illite, kaolinite and gibbsite either did not occur or was reduced because of the higher pH. No siderite precipitation was observed in any of the cases simulated.

Soil water pH was increased by about one unit, e.g. from 7.0 ~ 8.5 to 8.0 ~ 9.5 in the silt loam at the high infiltration rate. The extent of increase is comparable to what was observed in previous experimental studies, e.g. from 7 to 8.5 in Amann et al.^26^, from 3.55 to 4.69 ~ 5.18 in Dietzen et al.^6^, and from 5.0 ~ 6.5 to 6.5 ~ 7.5 in Haque et al.^22^. The increase in pH led to an increase for about one order of magnitude in acetate concentration, which provides a measure of the total dissolved organic carbon (DOC) in our simulations, because it is produced by cellulose degradation that is pH-dependent. Partial pressure of CO_2_ (${P}_{C{O}_{2}}$) shows a clear decreasing trend in the top layer as CO_2_ was consumed by mineral reactions, e.g. the precipitation of primarily magnesite and to a lesser extent calcite. The bottom section still shows an increasing trend as in the baseline case, but at values that are about one order of magnitude lower. Total inorganic carbon in the solution (TIC) is in general lower because of the transfer of carbon from solution to magnesite and calcite following the carbonation reaction, and is consistent with the lower ${P}_{C{O}_{2}}$ and higher pH.

### The impact of soil water content on forsterite weathering

**Figure 2:**
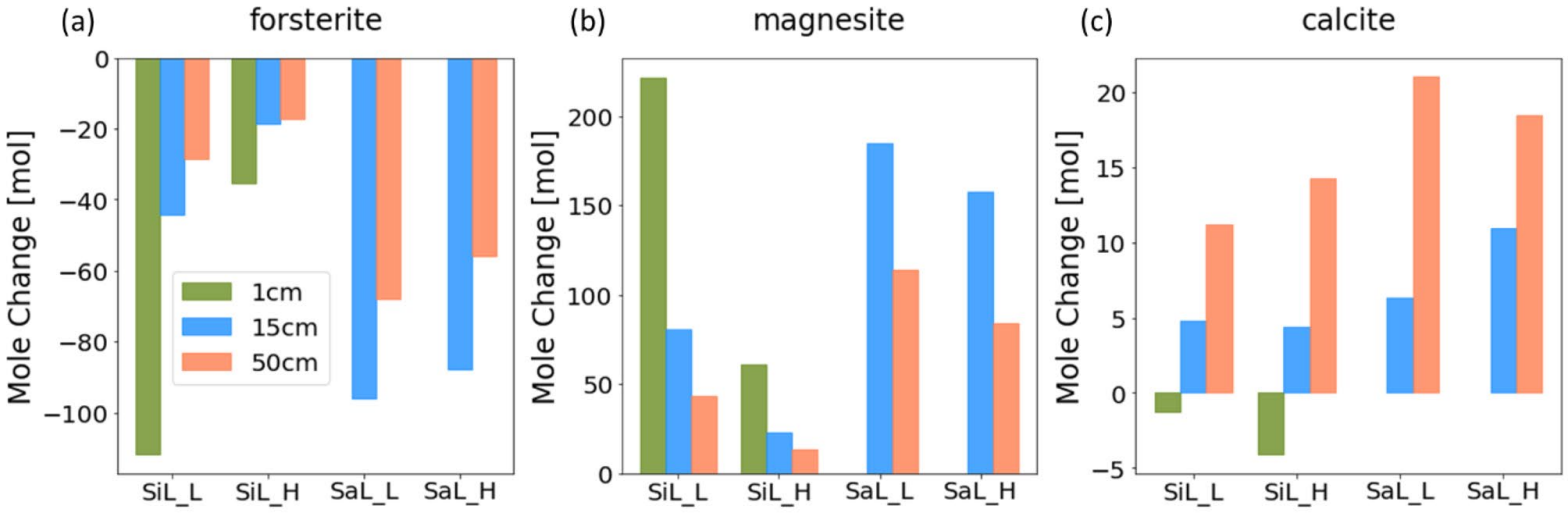
Mole changes of (**a**) forsterite, (**b**) magnesite, and (**c**) calcite for the silt loam (SiL) and the sandy loam (SaL) at the infiltration rate of 200 (_L) and 2000 (_H) mm/yr with different application scenarios for a specific surface area of 10m^2^/g for forsterite. Application depth: green – 1 cm, blue – 15 cm, coral – 50 cm (the total amount of the rock material applied is the same across the three application scenarios).

Figure 2 summarizes the total mole change of forsterite, magnesite, and calcite within the soil column by year five for the four water content cases with the largest forsterite surface area, and shows generally more weathering in well-drained soils (i.e. sandy loam) and at the low infiltration rate, i.e. under conditions generating lower water content.

For an application depth of 15 cm, more than 70% of the applied forsterite (~ 120 mol assuming a cross-section area of 1 m^2^) was weathered in the sandy loam. The weathering rate was slightly higher at the low infiltration rate. In contrast, about 35% and 15% of the applied forsterite was weathered in the silt loam at the low and high infiltration rate, respectively. The average dissolution rates ranged between ~ 5 × 10^–12^ mol/m^2^s to ~ 2 × 10^–11^ mol/m^2^s, in comparison to the intrinsic kinetic coefficients (2.29 × 10^–11^ mol/m^2^s for the neutral reaction mechanism and 1.41 × 10^–7^ mol/m^2^s for the acid reaction mechanism, Table S3). The amount of magnesite precipitation tracks forsterite dissolution, being about twice of the amount of forsterite following the stoichiometry of Mg in the two mineral phases. The variability of calcite precipitation across the four hydrological scenarios is similar to that of magnesite, except for the sandy loam cases, in which more precipitation was observed at the high infiltration rate. Calcium consumed by the precipitation reaction was primarily sourced from cation exchange. For instance, in the sandy loam at the low infiltration rate, the fraction of exchange sites occupied by Ca was reduced from ~ 0.4 to 0.2 in the topsoil where calcite precipitation occurred.

In comparison, with an application depth of 50 cm, forsterite weathering and magnesite precipitation show similar variations across soil types and infiltration rates, but at smaller amounts. There was more calcite precipitation, as precipitation also occurred in deeper sections of the soil column.

For the surficial application scenario (1 cm), forsterite weathering was higher in the silt loam at the low infiltration rate, showing complete weathering. In the sandy loam, the low water content and large forsterite surface area and thus fast forsterite dissolution created large local supersaturation with respect to amorphous silica. As a result, with the same simulation setup, amorphous silica precipitation led to a volume fraction change that is unrealistically high. These data points were hence excluded from the analyses.

Faster forsterite weathering under conditions generating a lower water content implies that atmospheric CO_2_ must be the dominant CO_2_ source in these simulations. CO_2_ available for the weathering reactions in the soil environment comes from three sources: CO_2_ that is dissolved in the infiltrating water, atmospheric CO_2_ transported into the soil matrix, and CO_2_ generated by organic matter (OM) decomposition. The first source accounts for a small fraction and is larger at the high infiltration rate. The second source is influenced by gas transport in our simulations, and the third source is controlled by the reaction network and soil chemistry. Given the reaction network considered, more biogenic CO_2_ should be generated at higher pH. In contrast, gas diffusion—which follows a power law relationship with respect to gas saturation—is faster when water content is lower (as in the sandy loam).

The CO_2_ profiles that show decreasing partial pressure in the top layer where forsterite was applied suggest a downward gas transport from the atmosphere. Even though the gradients in the lower section indicate an upward CO_2_ transport, the supply from soil decomposition was smaller than what would be expected based on the baseline simulations because of the elevated soil water pH and thus lower biogenic CO_2_.

The observation that with the high forsterite surface area, surficial application resulted in the fastest weathering rate is consistent with the conclusion that under these conditions, atmospheric CO_2_ was the major source driving the weathering reactions.

### The impact of soil chemistry on forsterite weathering

The observations above assumed that the functions of plant and microbial activities governing OM decomposition were neither promoted nor suppressed in response to the application of silicate rock materials and the subsequent changes in the soil chemistry, and the kinetic coefficients for the reaction network describing soil decomposition were fixed. However, studies have shown positive responses in functional and species richness of microbes to elemental and pH changes from enhanced rock weathering^44,45^. To investigate this potential scenario and the impact of the resulting soil chemistry on forsterite weathering, ${k}_{ox}$ was increased for the application depth of 15 cm.

**Figure 3:**
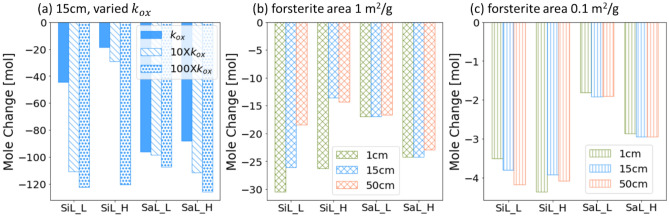
Mole changes of forsterite for the silt loam (SiL) and the sandy loam (SaL) at the infiltration rate of 200 (_L) and 2000 (_H) mm/yr (**a**) with different acetate oxidation rates (${k}_{ox}$) for a forsterite specific surface area of 10m^2^/g with an application depth of 15 cm, and with a forsterite specific surface area of (**b**) 1m^2^/g (diagonal grid pattern) and (**c**) 0.1 m^2^/g (vertical line pattern) with an application depth of 1 cm (green), 15 cm (blue), and 50 cm (blue).

Increasing ${k}_{ox}$ and thus CO_2_ partial pressure in the soil column resulted in an increase in forsterite weathering across all hydrological scenarios (Fig. 3a). The extent of increase is especially significant for the less well-drained soil (i.e. the silt loam), for which the availability of atmospheric CO_2_ was more constrained. With a 100× increase in ${k}_{ox}$, the applied forsterite was completely weathered by year five, except for the case of sandy loam at the low infiltration rate. This is likely caused by the fast gas transport under these conditions, which can limit the accumulation of CO_2_ in the soil column. In the sandy loam cases at the high infiltration rate, increased ${k}_{ox}$ resulted in higher rates of forsterite dissolution. This dependence on water flow indicates that in these cases, leaching of solutes rather than CO_2_ transport can be important in controlling the weathering rate^34^. In the silt loam at the low infiltration rate, increasing ${k}_{ox}$ by a factor of 10 resulted in weathering rate that is comparable with that of surficial application, indicating that CO_2_ from organic matter decomposition can make up for the constrained atmospheric CO_2_ supply in the deeper soil layers.

### The impact of surface area on forsterite weathering

The simulations presented above used a specific surface area of 10 m^2^/g, which corresponds to very fine grain sizes of the applied forsterite (~ 0.2 µ diameter assuming uniform spheres). This, however, would require considerable energy input (~ 700 MJ/tonne)^5^, which depending on the carbon intensity of the energy used may not be the optimal operational option. Here, we evaluate how variations in the specific surface area and thus grain size of the applied forsterite affects its weathering in the presence of transport constraint and microbe-mediated reactions. The average forsterite dissolution rates for simulations using different specific surface areas under all four hydrological scenarios ranged between 5 × 10^–13^ and 2 × 10^–11^ mol/m^2^s. They are comparable to previous pot experiments^19^, which used olivine grains that is composed of > 80% of forsterite with specific surface areas of 0.03–3 m^2^/g and measured dissolution rates between 4 × 10^–13^ and 3 × 10^–12^ mol/m^2^s.

When the specific surface area was increased from 0.1 to 1 m^2^/g (~ 2 and 20 µ grain size assuming uniform spheres, respectively), the weathering rates across all cases evaluated increased, but the extent of increase was not always proportional (Fig. 3b,c). For instance, in the sandy loam, weathering rate increased by a factor of 7.8–8.4 at the high infiltration rate, and by a factor of 8.7–9.4 at the low infiltration rate. This rate increase was much more moderate in the silt loam, being 3.4–6 and 4.4–8.3 at the high and low infiltration rate, respectively. Increasing the surface area of forsterite from 1 to 10 m^2^/g attenuated the weathering rate increase even further, with a factor of 1.2–1.4 in silt loam at the high infiltration rate and a factor of 4.1–5.7 in the sandy loam at the low infiltration rate. This brackets what has been reported in previous studies^46^.

With a specific surface area of 0.1 m^2^/g, forsterite weathering did not vary significantly across different application depths. This implies that CO_2_ transport was not limiting. Forsterite weathering was faster in the silt loam and at the high infiltration rate instead, as water is needed to keep the mineral saturation state low and thus the thermodynamic driving force high. As the specific surface area increased to 1 m^2^/g, the controls of CO_2_ transport became evident, showing similar variations across different application depths in the silt loam to the highest specific surface area simulations reported in previous sections. The increase in the relative magnitude of mineral reaction rates and transport rates, i.e. transport becomes increasingly limiting, also explains why the return of increasing surface area diminishes at higher surface area, as quantified above.

These observations highlight the necessity of considering the interplay between reaction kinetics and transport in the selection of operational parameters. At sites where transport of CO_2_ from the atmosphere is not or less limiting (e.g. the sandy loam), increasing surface area has a higher return in terms of improving weathering rate, while at sites where CO_2_ availability is already limiting (i.e. the silt loam), the improvement would be more moderate. In those cases, it would make more sense to reconsider the siting or to introduce microbial interventions rather than continuing with increasing the surface area, i.e. reducing grain sizes.

### Carbon accounting

**Figure 4:**
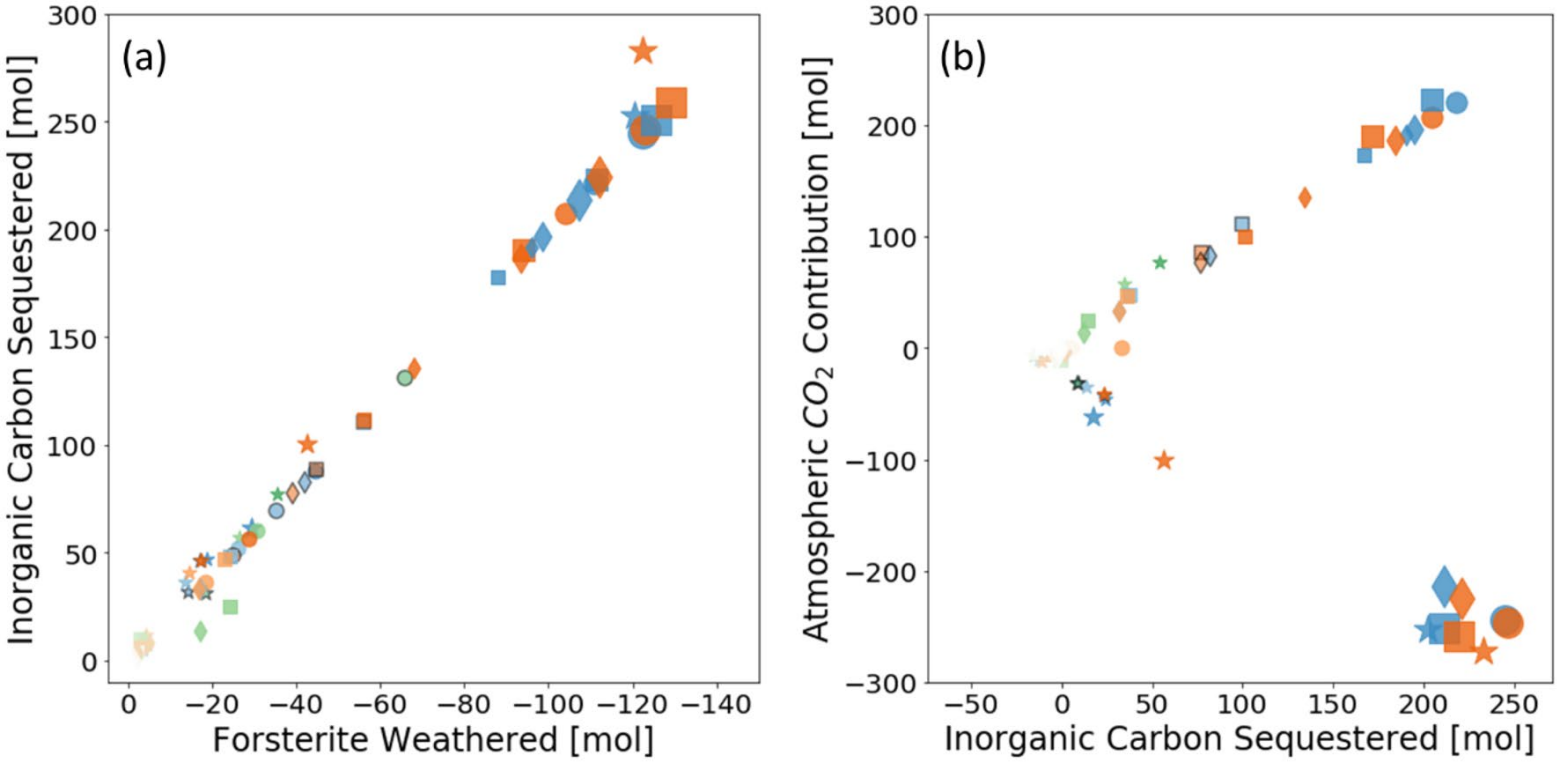
The amount of inorganic carbon sequestered [mol] in relation to (**a**) the amount of forsterite weathered [mol] and (**b**) the contribution from the atmosphere [mol]. The application depth is color coded: green – 1 cm, blue – 15 cm, and orange – 50 cm. The light and dark color corresponds to low and high $SS{A}_{for}$, respectively. The black edge indicates simulations with suppressed magnesite precipitation. The symbols indicate the hydrological scenarios: filled circle - silt loam at 200 mm/yr, filled asterisk - silt loam at 2000 mm/yr, filled diamond - sandy loam at 200 mm/yr, and filled square - sandy loam at 2000 mm/yr. The symbol size is enlarged by a factor of 2 and 3 for simulations using $10{k}_{ox}$ and $10{0k}_{ox}$, respectively.

Tracking the transport and fate of carbon is critical for carbon accounting and crediting. In our simulations, other than the exchange with the atmospheric CO_2_ at the top, all other carbon pools and fluxes were explicitly tracked. The simulations used a strict tolerance (10^–12^) to ensure mass conservation. Therefore, we can monitor the amount of carbon sequestered and the net carbon exchange with the atmosphere (Fig. 4a). Note that the organic carbon pool was highly simplified in our study and the changes in the organic carbon pool were not meant to capture sequestration via soil organic carbon, but to help break down the sources of carbon that was sequestered into the inorganic carbon pool.

The inorganic carbon (IC) sequestered measures the net change in the IC pool in the soil column and the amount of dissolved IC that enters the groundwater system at the bottom, and was calculated as the difference between the simulations with and without forsterite application. The amount of forsterite weathered and the amount of IC sequestered follows a linear relationship with a 1:2 ratio. This relationship is consistent with the stoichiometry of forsterite dissolution and magnesite precipitation.

4 $$M{g}_{2}Si{O}_{4}\left(s\right)+2C{O}_{2}\leftrightarrow 2MgC{O}_{3}+Si{O}_{2}\left(aq\right).$$

However, this ratio is not dependent on magnesite precipitation. Data points from simulations with suppressed magnesite precipitation (points with black edges in Fig. 4) follow the same trend, i.e. under these simulation conditions CO_3_^2–^ remains in the solution as the major form of carbon and as the major anion that balances the positive charges of the cations. With magnesite precipitation suppressed, Mg accumulation in the aqueous phase reduces the thermodynamic driving force for forsterite dissolution. Therefore, understanding the complex kinetics of magnesium carbonate precipitation under ambient conditions in the soil environment will be critical for evaluating ERW efficiency and engineering practices. IC sequestered is also attributed, to a lesser extent, to calcite precipitation and calcite dissolution avoided. The cumulative amount of carbon going into the groundwater was orders of magnitude lower than the amount contained in solid phases in our simulations, whereas under certain conditions CO_2_ may be stored primarily as HCO_3_^-^ in solution without precipitation^13^.

For most simulations, IC sequestered was primarily sourced from atmospheric CO_2_ (Fig. 4b). This is consistent with the observation that weathering rates in these cases were subject to CO_2_ transport limitation. In a few simulations with large application depths or with increased acetate oxidation (${k}_{ox}$), carbon was sequestered by reducing the biogenic CO_2_ emission that would otherwise occur without the application of forsterite (as noted by the negative signs). In these simulations, forsterite weathering removed local soil CO_2_.

The biotic effects, as reflected by elevated ${k}_{ox}$ values led to shifts in the amount of forsterite weathering and the relative contribution of atmospheric CO_2_, without affecting the relationship between the IC sequestered and the weathered forsterite. However, the relationship may vary if the biotic reactions change the partitioning of carbon between the solid phase and the aqueous phase, which have different stoichiometries. Moreover, future investigations are needed to fully assess the organic carbon sink in the soil and its relation with rock weathering, among which mineral-organic associations are a topic of great importance^47^.

### Implications for engineering design

While complete forsterite weathering (~ 120 mol) can occur within five years, which gives an equivalent carbon removal rate of ~ 2.3 kg CO_2_/m^2^/yr for a onetime application of ~ 16 kg/m^2^, much less carbon was sequestered under unfavorable conditions. Given that CO_2_ emission associated with crushing and milling of the rock materials can amount to ~ 10–25% of the CO_2_ sequestration capacity^27^, scenarios that result in less than 50 mol of IC sequestered after five years can hardly achieve carbon neutrality, let alone negative emissions. These are primarily simulations with the lowest specific surface area for forsterite. This is consistent with the previous observation that using grain size smaller than 10 µm is necessary to achieve significant CO_2_ removal^48^.

**Figure 5:**
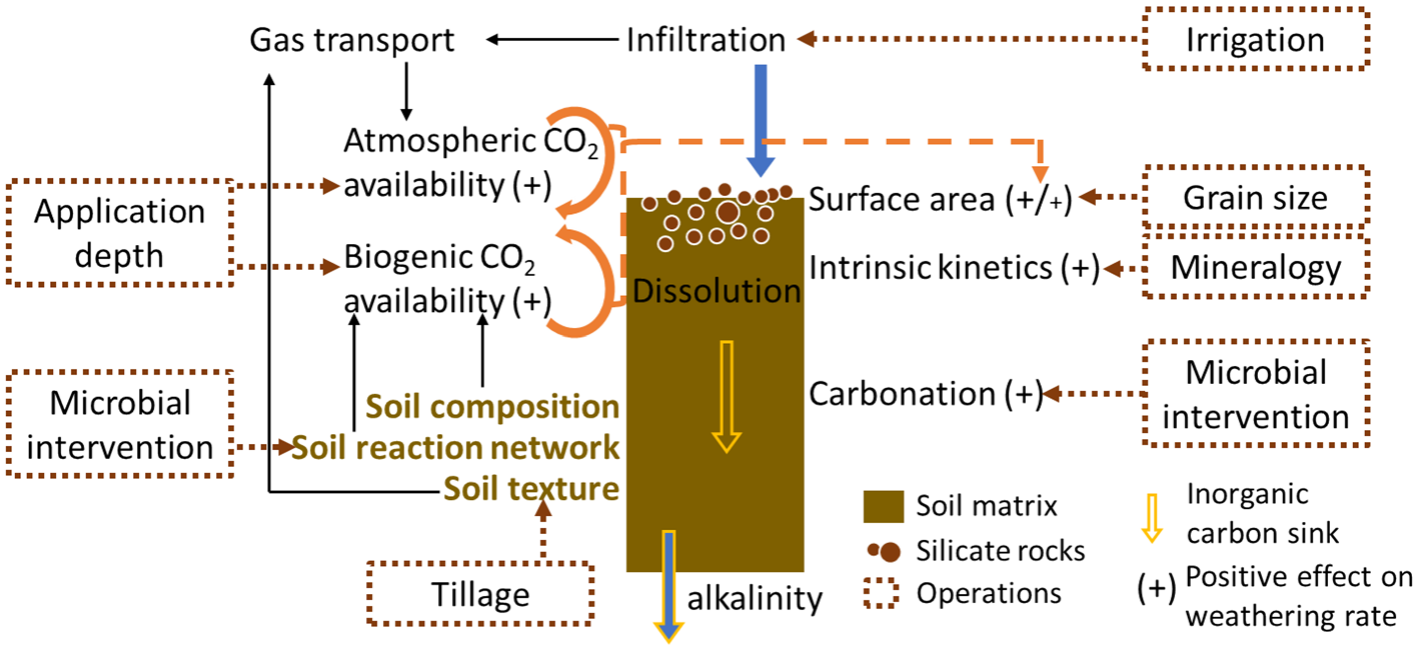
Schematic illustrating chemical and physical properties and processes that affect the efficiency of enhanced rock weathering in soils, and the impacts of engineering operations.

The weathering rate of a given rock material is controlled by the accessibility of the reactants, primarily the reactive rock surfaces and CO_2_ availability at the rock surface (Fig. 5). When atmospheric CO_2_ is the major source, the weathering rate is constrained by the smaller of the chemical reaction rate and the transport rate. Thus, while grinding the rock materials into fine grain sizes can enhance weathering, the extent of the increase is subject to the constraints on CO_2_ transport. When chemical reactions are relatively slow, increasing surface area by reducing grain size can boost the weathering rate significantly. Once the transport rate of reactants (e.g. CO_2_) to the rock materials or of the products away from the rock surfaces becomes limiting, increasing surface area has very limited effect on the weathering rate. It should also be noted that, as the grain size decreases, the energy penalty of grinding the particles down to finer sizes increases disproportionately^27^. Therefore, it is important to be able to identify an optimal grain size that balances the gain in weathering rate and thus carbon removal and the loss in energy due to comminution. The optimal grain size would depend on the crossing point of the chemical reaction rate and the transport rate. As illustrated in our simulations, for sites with high effectiveness of transporting CO_2_, the increase in weathering rate from increased surface area can be sustained for a wider range of surface area values, and thus the optimal grain size is likely to be smaller. Therefore, siting and operations that lead to effective CO_2_ transport will be important for ensuring ERW efficiency. For cases when grain size customization is impractical, other operations that relax the transport restrictions of CO_2_ at the sites should be considered, such as mixing or tillage that may change soil texture and hydraulic properties to improve CO_2_ transport. While our simulations only considered gas diffusion for CO_2_ transport, gas flow arising from complex hydrological processes provide another transport mechanism, and thus soil properties (e.g. textural heterogeneity), processes (e.g. transient flow and porosity–permeability change, see SI for porosity change observed in our simulations) and operations influencing gas flow need to be evaluated further.

Alternatively, high weathering rate can be achieved if local CO_2_ sources (i.e. CO_2_ from OM decomposition in the soil) can be tapped into, to relax the constraint of CO_2_ transport. This can potentially be realized by increasing the application depth to bring the reactive mineral closer to soil CO_2_. However, when OM decomposition cannot replenish CO_2_ consumed by the weathering reaction fast enough, a high weathering rate cannot be sustained. In this case, increasing the application depth becomes undesirable as it moves the rock material away from atmospheric CO_2_. In contrast, when local CO_2_ supply is ‘guaranteed’ by fast OM decomposition, increasing the application depth helps achieve the high weathering rate. It can also be expected that in these cases, the positive effect of increasing the surface area can be maintained. The observation that the optimal application depth can be highly variable suggests that decisions regarding application by airplane spreading versus slurry application or tillage should be based on site characteristics.

For local supply of soil CO_2_ to be consistently high, it is necessary that the microbial and plant activities respond positively to the application of rock materials and the resulting changes in soil chemistry. While there are still a lot of uncertainties regarding this topic, existing studies, though few, have shown promise^44,45^. In addition to providing CO_2_ for the weathering reactions, the biotic processes are also expected to enhance rock weathering and CO_2_ sequestration by increasing the availability of organic/chelating ligands that can bond with reaction products and by facilitating carbonation reactions^49,50^. Future studies that further our understanding of the underlying mechanisms associated with the interactions between silicate rock weathering and biotic factors and processes – such that the effects on weathering rates can be quantitatively evaluated for operation optimization – are in great need. Such studies will also help develop innovative approaches that enhance weathering rates via microbial intervention or other biotic processes.

It should be noted that there are other operational considerations in engineering designs, although not discussed here, warrant in-depth future investigations. One operational parameter that is anticipated to impact the achievable weathering rate in the field is the application rate and frequencies in addition to the application depth. Moreover, in regions where the mining industry is well established, grinding may not be costly, whereas transportation to the sites may account for a larger portion of the energy penalty^51^. In this case, spatial analyses are needed to optimize the match between suitable rock materials and the application sites.

## Conclusions

Our study aimed to elucidate the impacts of coupled biogeochemical reactions and transport processes on the efficiency of enhanced rock weathering (ERW) in soils and quantify the effects of a set of primary environmental and operational controls. Based on a reactive transport model considering both unsaturated soil hydrodynamics and a validated soil reaction network, our results provide important insights for the practices of enhanced silicate weathering as soil amendments.

ERW in soils can be an effective option for carbon sequestration, which removes CO_2_ either from the atmosphere directly, or by reducing soil related emissions. Its efficiency is promoted by conditions that maintain high CO_2_ availability, e.g. good connectivity with atmospheric CO_2_ and/or sufficient biogenic CO_2_ supply. Therefore, soil texture, composition, and reaction networks are important factors to consider for proper siting. It is also important to co-optimize the site conditions with engineering operations. For instance, the applied rocks should be placed close to the dominant CO_2_ source (e.g. via optimizing the application depth), and the optimal grain size for efficient CO_2_ removal is site-specific and reducing grain size should be considered only when other kinetic controls (e.g. of carbonation reaction) and CO_2_ supply are nonlimiting. We further recommend future research to improve understanding of biotic-abiotic interactions at their native scales, such that these effects can be quantified and properly assessed to ascertain ERW efficiency in soils.

## Supplementary Information

Supplementary Information.

## Data Availability

All input data and key modeling results have been included in the manuscript and the supplementary information. The model output can be made available upon reasonable request to the corresponding author.

## References

[1] McInerney D, Keller K (2008). Economically optimal risk reduction strategies in the face of uncertain climate thresholds. Clim. Change.

[2] National Academies of Sciences Engineering Medicine (2019). Negative Emissions Technologies and Reliable Sequestration: A Research Agenda.

[3] Minx JC (2018). Negative emissions—Part 1: Research landscape and synthesis. Environ. Res. Lett..

[4] Kelemen P, Benson SM, Pilorgé H, Psarras P, Wilcox J (2019). An overview of the status and challenges of CO_2_ storage in minerals and geological formations. Front. Clim..

[5] Beerling DJ (2020). Potential for large-scale CO_2_ removal via enhanced rock weathering with croplands. Nature.

[6] Dietzen C, Harrison R, Michelsen-Correa S (2018). Effectiveness of enhanced mineral weathering as a carbon sequestration tool and alternative to agricultural lime: An incubation experiment. Int. J. Greenh. Gas Control.

[7] Edwards DP (2017). Climate change mitigation: Potential benefits and pitfalls of enhanced rock weathering in tropical agriculture. Biol. Lett..

[8] Hartmann J (2013). Enhanced chemical weathering as a geoengineering strategy to reduce atmospheric carbon dioxide, supply nutrients, and mitigate ocean acidification. Rev. Geophys..

[9] Kantola IB, Masters MD, Beerling DJ, Long SP, DeLucia EH (2017). Potential of global croplands and bioenergy crops for climate change mitigation through deployment for enhanced weathering. Biol. Lett..

[10] Meysman FJR, Montserrat F (2017). Negative CO_2_ emissions via enhanced silicate weathering in coastal environments. Biol. Lett..

[11] Renforth P (2012). The potential of enhanced weathering in the UK. Int. J. Greenh. Gas Control.

[12] Strefler J, Amann T, Bauer N, Kriegler E, Hartmann J (2018). Potential and costs of carbon dioxide removal by enhanced weathering of rocks. Environ. Res. Lett..

[13] Köhler P, Hartmann J, Wolf-Gladrow Dieter A (2010). Geoengineering potential of artificially enhanced silicate weathering of olivine. Proc. Natl. Acad. Sci..

[14] Calabrese S (2022). Nano- to global-scale uncertainties in terrestrial enhanced weathering. Environ. Sci. Technol..

[15] Sanna A, Uibu M, Caramanna G, Kuusik R, Maroto-Valer MM (2014). A review of mineral carbonation technologies to sequester CO_2_. Chem. Soc. Rev..

[16] Vienne A (2022). Enhanced weathering using basalt rock powder: Carbon sequestration, co-benefits and risks in a mesocosm study with *Solanum*
*tuberosum*. Front. Clim..

[17] Goll DS (2021). Potential CO_2_ removal from enhanced weathering by ecosystem responses to powdered rock. Nat. Geosci..

[18] Lehmann J, Possinger A (2020). Removal of atmospheric CO_2_ by rock weathering holds promise for mitigating climate change. Nature.

[19] Renforth P, von Strandmann P, Henderson GM (2015). The dissolution of olivine added to soil: Implications for enhanced weathering. Appl. Geochem..

[20] Swoboda P, Doring TF, Hamer M (2022). Remineralizing soils? The agricultural usage of silicate rock powders: A review. Sci. Total Environ..

[21] de Oliveira Garcia W (2020). Impacts of enhanced weathering on biomass production for negative emission technologies and soil hydrology. Biogeosciences.

[22] Haque F, Santos R, Dutta A, Thimmanagari M, Chiang YW (2019). Co-benefits of wollastonite weathering in agriculture: CO_2_ sequestration and promoted plant growth. ACS Omega.

[23] Andrews MG, Taylor LL (2019). Combating climate change through enhanced weathering of agricultural soils. Elements.

[24] Lasaga AC (1995). Chemical Weathering Rates of Silicate Minerals.

[25] Oelkers EH, Declercq J, Saldi GD, Gislason SR, Schott J (2018). Olivine dissolution rates: A critical review. Chem. Geol..

[26] Amann T (2020). Enhanced weathering and related element fluxes—A cropland mesocosm approach. Biogeosciences.

[27] Moosdorf N, Renforth P, Hartmann J (2014). Carbon dioxide efficiency of terrestrial enhanced weathering. Environ. Sci. Technol..

[28] Cipolla G, Calabrese S, Porporato A, Noto LV (2022). Effects of precipitation seasonality, irrigation, vegetation cycle and soil type on enhanced weathering - modeling of cropland case studies across four sites. Biogeosciences.

[29] Arora B (2016). Influence of hydrological, biogeochemical and temperature transients on subsurface carbon fluxes in a flood plain environment. Biogeochemistry.

[30] Waterhouse H (2021). Influence of agricultural managed aquifer recharge (AgMAR) and stratigraphic heterogeneities on nitrate reduction in the deep subsurface. Water Resour. Res..

[31] Baker, S. E. et al. Getting to neutral: Options for negative carbon emissions in California. 10.2172/1597217 (2020).

[32] Xu T (2006). TOUGHREACT—A simulation program for non-isothermal multiphase reactive geochemical transport in variably saturated geologic media: Applications to geothermal injectivity and CO_2_ geological sequestration. Comput. Geosci..

[33] Xu, T., Sonnenthal, E., Spycher, N. & Pruess, K. TOUGHREACT user’s guide: A simulation program for non-isothermal multiphase reactive geochemical transport in variable saturated geologic media. 10.2172/834237 (2004).

[34] Cipolla G, Calabrese S, Noto LV, Porporato A (2021). The role of hydrology on enhanced weathering for carbon sequestration II. From hydroclimatic scenarios to carbon-sequestration efficiencies. Adv. Water Resour..

[35] McCutcheon J (2019). Carbon sequestration in biogenic magnesite and other magnesium carbonate minerals. Environ. Sci. Technol..

[36] Power IM, Kenward PA, Dipple GM, Raudsepp M (2017). Room temperature magnesite precipitation. Cryst. Growth Des..

[37] Palandri, J. L. *A compilation of rate parameters of water-mineral interaction kinetics for application to geochemical modeling [electronic resource]/by James L. Palandri and Yousif K. Kharaka; prepared in cooperation with the National Energy Technology Laboratory, United States Department of Energy*. (U.S. Dept. of the Interior, U.S. Geological Survey, 2004).

[38] Tuller M, Or D (2005). Water films and scaling of soil characteristic curves at low water contents. Water Resour. Res..

[39] Appelo CAJPD (2005). Geochemistry, Groundwater and Pollution.

[40] Wang Y (2018). Depth-dependent greenhouse gas production and consumption in an upland cropping system in northern China. Geoderma.

[41] Haque F, Santos RM, Chiang YW (2020). Optimizing inorganic carbon sequestration and crop yield with wollastonite soil amendment in a microplot study. Front. Plant Sci..

[42] Brantley SL, Mellott NP (2000). Surface area and porosity of primary silicate minerals. Am. Miner..

[43] Giammar DE, Bruant RG, Peters CA (2005). Forsterite dissolution and magnesite precipitation at conditions relevant for deep saline aquifer storage and sequestration of carbon dioxide. Chem. Geol..

[44] Balland-Bolou-Bi C, Poszwa A (2012). Effect of calco-magnesian amendment on the mineral weathering abilities of bacterial communities in acidic and silicate-rich soils. Soil Biol. Biochem..

[45] Verbruggen E, Struyf E, Vicca S (2021). Can arbuscular mycorrhizal fungi speed up carbon sequestration by enhanced weathering?. Plants People Planet.

[46] Lewis AL (2021). Effects of mineralogy, chemistry and physical properties of basalts on carbon capture potential and plant-nutrient element release via enhanced weathering. Appl. Geochem..

[47] Kleber M (2015). Chapter one—Mineraœ organic associations: Formation, properties, and relevance in soil environments. Adv. Agron..

[48] Rinder T, von Hagke C (2021). The influence of particle size on the potential of enhanced basalt weathering for carbon dioxide removal—Insights from a regional assessment. J. Clean. Prod..

[49] Wu S (2019). Organic matter amendment and plant colonization drive mineral weathering, organic carbon sequestration, and water-stable aggregation in magnetite fe ore tailings. Environ. Sci. Technol..

[50] Thorley RMS, Taylor LL, Banwart SA, Leake JR, Beerling DJ (2015). The role of forest trees and their mycorrhizal fungi in carbonate rock weathering and its significance for global carbon cycling. Plant Cell Environ..

[51] Lefebvre D (2019). Assessing the potential of soil carbonation and enhanced weathering to sequester atmospheric CO _2_ , through life cycle assessment: A case study for Sao Paulo State, Brazil. J. Clean. Prod..

