# Supplementary material for: The environmental controls on efficiency of enhanced rock weathering in soils
Source: Sci Rep. 2023 Jun 16;13:9765. doi: 10.1038/s41598-023-36113-4 (PMC10275906; doi:10.1038/s41598-023-36113-4)
Supplement: Supplementary file 1 — Supplementary Information. [file 41598_2023_36113_MOESM1_ESM.docx]

**Supplementary Information**

**The environmental controls on efficiency Enhanced rock weathering in soils**

Hang Deng^1*^, Eric Sonnenthal^2^, Bhavna Arora^2^, Hanna Breunig^3^, Eoin Brodie^2^, Markus Kleber^4^, Nicolas Spycher^2^, Peter Nico^2^

1. Department of Energy and Resources Engineering, College of Engineering, Peking University, Beijing, China, 100871
2. Earth and Environmental Sciences Area, Lawrence Berkeley National Laboratory, Berkeley, California, U.S.A., 94720
3. Energy Analysis and Environmental Impacts Division, Energy Technology Area, Lawrence Berkeley National Laboratory, Berkeley, California, U.S.A., 94720
4. Department of Crop and Soil Science, College of Agricultural Sciences, Oregon State University, Corvallis, Oregon, U.S.A., 97331

*Corresponding author: hangdeng@pku.edu.cn

**Table of Contents**

[1.Reactive transport model 2](#_Toc119943813)

[2. Simulation setup 3](#_Toc119943814)

[3. Vertical profiles of mineral phases 8](#_Toc119943815)

[4. Porosity Change 8](#_Toc119943816)

[References 8](#_Toc119943817)

**List of Figures**

[Figure S1. Steady state depth profiles of water saturation in the silt loam and sandy loam at an infiltration rate of 200 and 2000 mm/yr. 4](#_Toc119943882)

[Figure S2. Vertical profiles at year five for an application of 15 cm forsterite with 10 m^2^/g surface area for both soil types at both infiltration rates. Changes in the volume fractions of (a) montmorillonite, (b) k-feldspar, and (c) cellulose. 8](#_Toc119943883)

**List of Tables**

[Table S1. Hydraulic properties of a silt loam and a sandy loam, following ^4^. 4](#_Toc119943896)

[Table S2. Soil composition for the silt loam and sandy loam used in our study following the volume fractions reported in ^4^. Volume fraction of quartz was adjusted. 4](#_Toc119943897)

[Table S3. Primary and secondary minerals reactions and parameters included in our study. There kinetic coefficients and other coefficients and other coefficients used in eqn(S10) are taken from ^5^. 4](#_Toc119943898)

[Table S4. Microbe-mediated reactions used in our study, following ^4, 6^, except for the coefficient noted by the superscript a, which is referred to as $k_{ox}$ in the text. 6](#_Toc119943899)

[Table S5. composition of the initial and infiltrating water, adjusted based on ^4^. a – at equilibrium with calcite, b – at equilibrium with atmospheric CO_2_, c – at equilibrium with atmospheric O_2_, d – at equilibrium with K-feldspar, e – at equilibrium with ferrihydrite, and f – at equilibrium with cellulose. 6](#_Toc119943900)

[Table S6. A summary of the simulation scenarios. The columns are the four combinations of hydraulic conditions, and the rows are rock application scenarios. For each hydraulic condition and rock application, the specific surface areas for forsterite ($SSA_{for}$) and $k_{ox}$. 7](#_Toc119943901)

## 1. Reactive transport model

The governing equation for the hydrodynamics is given as:

$\frac{\partial(\phi S_{l}\rho_{l})}{\partial t}=-\nabla\left( \rho_{l}u_{l} \right)$ (S1)

where $\phi$ is the porosity, $\rho_{l}$ [kg/m^3^] is the density for water, and $S_{l}$ is the water saturation. $u_{l}$ is the Darcy velocity [m/s], which can be calculated from:

$u_{l}=-k\frac{k_{r,l}}{\mu_{l}}\left( \nabla P_{l}-\rho_{l}gz \right)$ (S2)

where $k$ is the permeability, $k_{r,l}$ is the relative permeability for water, $\mu_{l}$ is the water viscosity, $P_{l}$ is the water pressure [Pa], $g$ is the gravitational acceleration [m/s^2^], and $z$ is the depth. The relative permeability is given by the van Genuchten-Mualem model:

$k_{r,l}=\left\{ \begin{aligned} \sqrt{S^{*}}\left\{ 1-\left( 1-{S^{*}}^{\frac{1}{m}} \right)^{m} \right\}^{2} S_{l}<S_{ls} \\ 1 S_{l}\geq S_{ls} \end{aligned} \right.$ (S3)

$S^{*}=(S_{l}-S_{lr})/(S_{ls}-S_{lr})$ (S4)

where $S_{lr}$ is the residual water saturation, and $S_{ls}$ is the maximum saturation, which in this case is the same as $\phi$. $S_{lr}$ and $m$ are parameters that are dependent on the soil texture.

Capillary pressure ($P_{cap}$) is the difference between $P_{l}$ and the gas pressure ($P_{g}$), and in the van Genuchten model, it is calculated as:

$P_{cap}=-\frac{\alpha}{\rho_{l}g}\left( {S^{*}}^{-\frac{1}{m}}-1 \right)^{1-m}$ (S5)

where $\alpha$ is also a parameter that varies with the hydraulic properties of the soil.

For a given chemical component $i$ in the aqueous phase, its concentration ($C_{il}$) is given by the mass conservation equation:

$\frac{\partial(\phi S_{l}C_{il})}{\partial t}=-\nabla\left( u_{l}C_{il}-\left( \tau_{l}\phi S_{l}D_{l} \right)\nabla C_{il} \right)+q_{i,l}+q_{i,s}+q_{i,g}$ (S6)

where $q_{i,l}$, $q_{i,s}$, and $q_{i,g}$ are the source/sink term in the liquid, solid and gas phase, respectively. $D_{l}$ is the diffusion coefficient [m^2^/s], and $\tau$ is tortuosity given by ^1^:

$\tau_{l}=\phi^{\frac{1}{3}}S_{l}^{\frac{7}{3}}$ (S7)

The governing equation for the gas phase follows a similar form as eqn(S6), but with parameters for the gas phase instead of the liquid phase, and neglecting the advective term in eqn(S6). The tortuosity is calculated from gas saturation (as in eqn(S7), and the diffusion coefficient of gas was calculated by eqn (S8).

$D_{g}=\frac{RT}{3\sqrt{2}\pi PN_{A}d_{m}^{2}}\sqrt{\frac{8RT}{\pi M}}$ (S8)

where $N_{A}$ is the Avogadro’s number, $d_{m}$ and $M$ are the diameter and weight of the gas molecular, $R$ is the ideal gas constant, and $T$ is the temperature, which is 18 °C for our simulations. The concentration is defined based on the partial pressure of the gas species $j$ ($P_{g,j}$) as follows:

$C_{g,j}=\frac{P_{g,j}\phi S_{g}\rho_{g}}{MW_{g}P_{g}{10}^{-5}}$ (S9)

where $\rho_{g}$ and $MW_{g}$ are the density and molecular weight of the carrier gas, which is air in this case, respectively.

The geochemical reactions are captured by the source/sink terms. Specifically, kinetic mineral reactions are calculated based on the Transition State Theory ^2^, and the reaction rate for mineral $n$ is given as

$R_{n}=A_{n}\sum{(k}_{n,j}e^{-\frac{{E_{a}}_{j}}{RT}}\prod a_{j}^{n_{j}})\left( 1-\Omega_{n}^{\theta} \right)^{\eta}$ (S10)

where$A_{n}$ is the surface area, $k_{n,j}$ is the kinetic coefficient of a given parallel reaction pathway, $E_{a}$ is the activation energy, $R$ is the ideal gas constant, $T$ is the temperature, and $a_{j}$ and $n_{j}$ are the activity of the catalytic/inhibitory species and the corresponding exponent, respectively. $\Omega_{n}$ is the saturation state with respect to the mineral, and $\theta$ and $\eta$ are coefficients depending on the reaction mechanisms and are one unless specified otherwise.

Another group of reactions that are important in the soil environment is the microbial mediated reactions, which is implemented in our simulations by a general rate law that considers multiple mechanisms:

$R_{m}=\mu_{max}\prod\frac{C_{s,i}}{K_{s,i}+C_{s,i}}\prod\frac{K_{I,j}}{K_{I,j}+C_{I,j}}$ (S11)

where $\mu_{max}$ is the maximum rate of reaction, $C_{s,i}$ and $C_{I,j}$ are the concentrations of the Monod species and the inhibitory species, respectively, and $K_{s,i}$ and $K_{I,j}$ are the corresponding half saturation coefficients. In this formulation, the reaction rate is independent of bacterial growth and decay, as bacterial cells are not explicitly tracked in the simulations.

Cation exchange was also included following the Gaines-Thomas convention.

$K_{Na/M}^{*}=\frac{X_{Na}\cdot a_{M}^{\frac{1}{Z_{M}}}}{X_{M}^{\frac{1}{Z_{M}}}\cdot X_{Na}}$ (S12)

where $M$ is another cation (e.g., $K^{+}$, ${Ca}^{2+}$, ${Mg}^{2+}$) , and $Z_{M}$ is its charge, $X_{M}$ is the equivalent fraction of the number of exchange sites occupied by the species. The exchange coefficients ($K^{*}$) are 0.2, 0.4, and 0.5, for $K^{+}$, ${Ca}^{2+}$, and ${Mg}^{2+}$, respectively ^3^.

## 2. Simulation setup

Hydraulic properties (Table S1) and soil compositions (Table S2) of the two soils used in the simulations, kinetic parameters of the mineral reactions (Table S3) and the microbe-mediated reactions (Table S4) are provided below. Figure S1 shows the water content profiles of the four hydrological scenarios. The compositions of the “initial” and infiltrating water are summarized in Table S5. The “initial” water composition represents groundwater at the water table (fixed composition with time) as well as the modeled initial composition of pore water in the vadose zone before it is flushed by infiltration. Table S6 summarizes the simulations performed in our study.

Table S1. Hydraulic properties of a silt loam and a sandy loam, following ^4^.

|  | Sand [%] | Silt [%] | Clay [%] | k [m^2^] | n | $\alpha$ [cm^-1^] | $S_{lr}$ | $S_{ls} (\phi)$ |
| --- | --- | --- | --- | --- | --- | --- | --- | --- |
| Silt loam | 37 | 53 | 10 | 1.30E-13 | 1.41 | 0.02 | 0.067 | 0.46 |
| Sandy loam | 66 | 28 | 6 | 1.30E-12 | 1.89 | 0.075 | 0.065 | 0.41 |


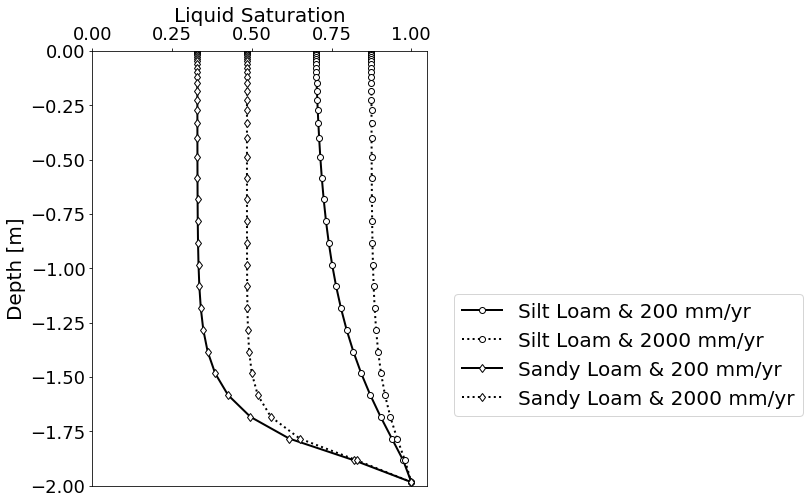


Figure S1. Steady state depth profiles of water saturation in the silt loam and sandy loam at an infiltration rate of 200 and 2000 mm/yr.

Table S2. Soil composition for the silt loam and sandy loam used in our study following the volume fractions reported in ^4^. Volume fraction of quartz was adjusted.

|  | Cellulose | K-feldspar | Montmorillonite- Na | Calcite | Quartz | Ferrihydrite |
| --- | --- | --- | --- | --- | --- | --- |
| **Silt Loam** | 0.08 | 0.09 | 0.17 | 0.008 | 0.6497 | 0.0023 |
| **Sandy Loam** | 0.03 | 0.09 | 0.08 | 0.035 | 0.7632 | 0.0018 |

Table S3. Primary and secondary minerals reactions and parameters included in our study. There kinetic coefficients and other coefficients and other coefficients used in eqn(S10) are taken from ^5^. In the column of ‘species: n’: ‘H^+^’ with positive n values suggest that the corresponding entry is for the acid reaction mechanism, ‘OH^-^’ with positive n values or ‘H^+^’ with negative n values are for the base reaction mechanism, ‘CO_3_^2-^’ indicates that the corresponding kinetic coefficient accounts for the catalytic mechanism of carbonate species, otherwise, the corresponding Log $k$ values are for the neutral reaction mechanism.

| Minerals | | Log $k$ (25$℃$)  (mol/m^2^s) | $E_{a}$ (kJ/mol) | Species: $n$ | Surface area | Initial volume fraction |
| --- | --- | --- | --- | --- | --- | --- |
| Primary | Cellulose | Equilibrium | | | | See Table 2 |
|  | K-feldspar | 3.89$\times$10^-13^ | 38.00 |  | 1.5$\times$10^7^  m^2^ soil/m^3^ soil | See Table 2 |
|  |  | 8.71$\times$10^-11^ | 51.7 | $H^{+}$: 0.5 |  |  |
|  |  | 6.31$\times$10^-22^ | 94.1 | $H^{+}$: -0.823 |  |  |
|  | Montmorillonite-Na | $1.66\times$10^-13^ | 35.00 |  | 1.5$\times$10^7^  m^2^ soil/m^3^ soil | See Table 2 |
|  |  | $1.95\times$10^-13^ | 48.0 | $H^{+}$: 0.34 |  |  |
|  |  | 3.02$\times$10^-17^ | 58.9 | $H^{+}$: -0.4 |  |  |
|  | Calcite | 1.55$\times$10^-6^ | 23.50 |  | 1.5$\times$10^7^  m^2^ soil/m^3^ soil | See Table 2 |
|  |  | 5.01$\times$10^-1^ | 14.4 | $H^{+}$: 1.0 |  |  |
|  |  | 3.31$\times$10^-4^ | 35.4 | $CO_{3}^{2-}$: 1.0 |  |  |
|  | Quartz | 4.57$\times$10^-14^ | 90.1 |  | 1.5$\times$10^7^  m^2^ soil/m^3^ soil | See Table 2 |
|  | Ferrihydrite | 1.15$\times$10^-7^ | 86.5 |  | 1.5$\times$10^7^  m^2^ soil/m^3^ soil | See Table 2 |
|  | Forsterite | 2.29$\times$10^-11^ | 79.0 |  | 0.1-10 m^2^/g | See Table 2 |
|  |  | 1.41$\times$10^-7^ | 67.2 | $H^{+}$: 0.47 |  |  |
| Secondary | Amorphous SiO_2_ | 3.80$\times$10^-10^ | 49.8 |  | 1.5$\times$10^7^  m^2^ soil/m^3^ soil | 1.0$\times$10^-6^ |
|  | Siderite | 2.1$\times$10^-9^ | 56.00 |  | 1.5$\times$10^7^  m^2^ soil/m^3^ soil | 1.0$\times$10^-6^ |
|  |  | 5.96$\times$10^-6^ | 56.0 | $H^{+}$: 0.6 |  |  |
|  | Illite | 3.30$\times$10^-17^ | 35.00 |  | 1.5$\times$10^7^  m^2^ soil/m^3^ soil | 1.0$\times$10^-6^ |
|  |  | $9.8\times$10^-12^ | 36.00 | $H^{+}$: 0.52 |  |  |
|  |  | 3.10$\times$10^-12^ | 48.00 | $OH^{-}$: 0.38 |  |  |
|  | Kaolinite | 6.61$\times$10^-14^ | 22.20 |  | 1.5$\times$10^7^  m^2^ soil/m^3^ soil | 1.0$\times$10^-6^ |
|  |  | 4.90$\times$10^-12^ | 65.9 | $H^{+}$: 0.777 |  |  |
|  |  | 8.91$\times$10^-18^ | 17.9 | $H^{+}$:-0.472 |  |  |
|  | Gibbsite | 3.16$\times$10^-12^ | 61.20 |  | 1.5$\times$10^7^  m^2^ soil/m^3^ soil | 1.0$\times$10^-6^ |
|  |  | 2.24$\times$10^-8^ | 47.5 | $H^{+}$: 0.992 |  |  |
|  |  | 2.24$\times$10^-17^ | 80.1 | $H^{+}$:-0.784 |  |  |
|  | Magnesite | 4.57$\times$10^-10^ | 23.50 |  | 10 m^2^/g | 1.0$\times$10^-6^ |
|  |  | 4.17$\times$10^-7^ | 14.4 | $H^{+}$: 1.0 |  |  |
|  |  | 6.03$\times$10^-6^ | 62.8 | $CO_{3}^{2-}$: 1.0 |  |  |

Table S4. Microbe-mediated reactions used in our study, following ^4, 6^, except for the coefficient noted by the superscript a, which is referred to as $k_{ox}$ in the text.

| Reaction | Log K (25$℃$) | $\mu_{max}$ (mol/L/s) | $K_{s}$(mol/L) | $K_{I}$(mol/L) |
| --- | --- | --- | --- | --- |
| $CH_{3}COO^{-}+2O_{2}\to2HCO_{3}^{-}+H^{+}$ | 146.76 | 1.0$\times$10^-10 a^ | $O_{2}$: 2.41$\times$10^-5^ |  |
| $CH_{3}COO^{-}+4NO_{3}^{-}\to2HCO_{3}^{-}+4NO_{2}^{-}+H^{+}$ | 89.04 | 2.78$\times$10^-10^ | ${NO}_{3}^{-}$: 1.13$\times$10^-4^ | $O_{2}$: 3.22$\times$10^-7^ |
| $CH_{3}COO^{-}+2.667NO_{2}^{-}+1.667H^{+}\to2HCO_{3}^{-}+1.33N_{2}+1.33H_{2}O$ | 200.52 | 3.47$\times$10^-8^ | ${NO}_{2}^{-}$: 1.13$\times$10^-4^ | $O_{2}$: 3.22$\times$10^-7^ |
| $NH_{3}\left( aq \right)+2O_{2}\to NO_{3}^{-}+H_{2}O+H^{+}$ | 62.23 | $5.27\times$10^-2^ | $NH_{3}$: 1.48$\times$10^-5^  $O_{2}$: 2.41$\times$10^-5^ |  |
| $CH_{3}COO^{-}+8Fe^{3+}+4H_{2}O\to8Fe^{2+}+ 2HCO_{3}^{-}+9H^{+}$ | 79.00 | 1.0$\times$10^-14^ |  | $O_{2}$: 3.22$\times$10^-7^  ${NO}_{3}^{-}$: 1.0$\times$10^-7^ |
| $Fe^{2+}+0.2NO_{3}^{-}+1.2H^{+}\to Fe^{3+}+0.1N_{2}+0.6H_{2}O$ | -7.32 | 7.0$\times$10^-10^ | $Fe^{2+}$: 1.0$\times$10^-5^  ${NO}_{3}^{-}$: 1.13$\times$10^-4^ | $O_{2}$: 3.22$\times$10^-7^  ${NO}_{3}^{-}$: 1.0$\times$10^-7^ |
| $CH_{3}COO^{-}+SO_{4}^{2-}\to2HCO_{3}^{-}+HS^{-}$ | 8.40 | 3.0$\times$10^-12^ | $SO_{4}^{2-}$: 1.0$\times$10^-3^ | $O_{2}$: 3.22$\times$10^-7^  ${NO}_{3}^{-}$: 1.0$\times$10^-7^  $Fe^{3+}$: 1.0$\times$10^-12^ |
| $HS^{-}+1.6NO_{3}^{-}+0.6H^{+}\to SO_{4}^{2-}+0.8N_{2}+0.8H_{2}O$ | 11.52 | 7.0$\times$10^-10^ | $HS^{-}$: 1.0$\times$10^-5^  ${NO}_{3}^{-}$: 1.13$\times$10^-4^ | $O_{2}$: 3.22$\times$10^-7^ |

Table S5. composition of the initial and infiltrating water, adjusted based on ^4^. a – at equilibrium with calcite, b – at equilibrium with atmospheric CO_2_, c – at equilibrium with atmospheric O_2_, d – at equilibrium with K-feldspar, e – at equilibrium with ferrihydrite, and f – at equilibrium with cellulose.

|  | Initial water | Infiltrating water |
| --- | --- | --- |
| pH | 7.5 | 6.63 |
| ${SiO}_{2}(aq)$ | 6.83E-04 | 6.83E-06 |
| $Na^{+}$ | 1.83E-03 | 1.83E-05 |
| $K^{+}$ | 7.42E-05 | 7.42E-07 |
| $Ca^{2+}$ | 9.74E-04 | 9.74E-06 |
| ${Mg}^{2+}$ | 3.50E-04 | 3.50E-06 |
| $HCO_{3}^{-}$ | 5.20E-03 ^a^ | 4.57E-05 ^b^ |
| *log*$P_{CO_{2}}$ | -2.08 | -3.4 |
| $SO_{4}^{2-}$ | 1.25E-04 | 1.25E-06 |
| $Cl^{-}$ | 6.77E-04 | 6.77E-06 |
| $O_{2}(aq)$ | 2.85E-04 ^c^ | 2.85E-04 ^c^ |
| $HS^{-}$ | 1.00E-20 | 1.00E-20 |
| $NO_{3}^{-}$ | 7.14E-04 | 7.14E-06 |
| $NO_{2}^{-}$ | 9.35E-07 | 9.35E-09 |
| $N_{2}(aq)$ | 1.00E-20 | 1.00E-10 |
| $NH_{3}(aq)$ | 1.00E-08 | 1.00E-09 |
| $Al^{3+}$ | 6.77E-10 ^d^ | 1.00E-20 |
| ${Fe}^{3+}$ | 9.00E-10 ^e^ | 1.00E-20 |
| ${Fe}^{2+}$ | 8.30E-20 | 8.30E-20 |
| ${Ba}^{2+}$ | 4.15E-07 | 1.00E-20 |
| ${Sr}^{2+}$ | 5.17E-06 | 1.00E-20 |
| Acetate | 1.14E-05 ^f^ | 1.19E-07 |

Table S6. A summary of the simulation scenarios. The columns are the four combinations of hydraulic conditions, and the rows are rock application scenarios. For each hydraulic condition and rock application, the specific surface areas for forsterite ($SSA_{for}$) and $k_{ox}$.

|  | Silt loam | | | | Sandy loam | | | |
| --- | --- | --- | --- | --- | --- | --- | --- | --- |
|  | 200 mm/yr | | 2000 mm/yr | | 200 mm/yr | | 2000 mm/yr | |
| Baseline  (no rock application) |  | $k_{ox}$,${10k}_{ox}$*,*${100k}_{ox}$ |  | $k_{ox}$,${10k}_{ox}$*,*${100k}_{ox}$ |  | $k_{ox}$,${10k}_{ox}$*,*${100k}_{ox}$ |  | $k_{ox}$,${10k}_{ox}$*,*${100k}_{ox}$ |
| Forsterite application 1cm | $SSA_{for}$  $0.1 m^{2}/g$ | $k_{ox}$ | $SSA_{for}$  $0.1 m^{2}/g$ | $k_{ox}$ | $SSA_{for}$  $0.1 m^{2}/g$ | $k_{ox}$ | $SSA_{for}$  $0.1 m^{2}/g$ | $k_{ox}$ |
|  | $1 m^{2}/g$ | $k_{ox}$ | $1 m^{2}/g$ | $k_{ox}$ | $1 m^{2}/g$ | $k_{ox}$ | $1 m^{2}/g$ | $k_{ox}$ |
|  | $10 m^{2}/g$ | $k_{ox}$ | $10 m^{2}/g$ | $k_{ox}$ | $10 m^{2}/g$ | $k_{ox}$ | $10 m^{2}/g$ | $k_{ox}$ |
| Forsterite application 15cm | $SSA_{for}$  $0.1 m^{2}/g$ | $k_{ox}$ | $SSA_{for}$  $0.1 m^{2}/g$ | $k_{ox}$ | $SSA_{for}$  $0.1 m^{2}/g$ | $k_{ox}$ | $SSA_{for}$  $0.1 m^{2}/g$ | $k_{ox}$ |
|  | $1 m^{2}/g$ | $k_{ox}$ | $1 m^{2}/g$ | $k_{ox}$ | $1 m^{2}/g$ | $k_{ox}$ | $1 m^{2}/g$ | $k_{ox}$ |
|  | $10 m^{2}/g$ | $k_{ox}$,${10k}_{ox}$*,*${100k}_{ox}$ | $10 m^{2}/g$ | $k_{ox}$,${10k}_{ox}$*,*${100k}_{ox}$ | $10 m^{2}/g$ | $k_{ox}$,${10k}_{ox}$*,*${100k}_{ox}$ | $10 m^{2}/g$ | $k_{ox}$,${10k}_{ox}$*,*${100k}_{ox}$ |
| Forsterite application 50cm | $SSA_{for}$  $0.1 m^{2}/g$ | $k_{ox}$ | $SSA_{for}$  $0.1 m^{2}/g$ | $k_{ox}$ | $SSA_{for}$  $0.1 m^{2}/g$ | $k_{ox}$ | $SSA_{for}$  $0.1 m^{2}/g$ | $k_{ox}$ |
|  | $1 m^{2}/g$ | $k_{ox}$ | $1 m^{2}/g$ | $k_{ox}$ | $1 m^{2}/g$ | $k_{ox}$ | $1 m^{2}/g$ | $k_{ox}$ |
|  | $10 m^{2}/g$ | $k_{ox}$ | $10 m^{2}/g$ | $k_{ox}$ | $10 m^{2}/g$ | $k_{ox}$ | $10 m^{2}/g$ | $k_{ox}$ |

## 3. Vertical profiles of mineral phases


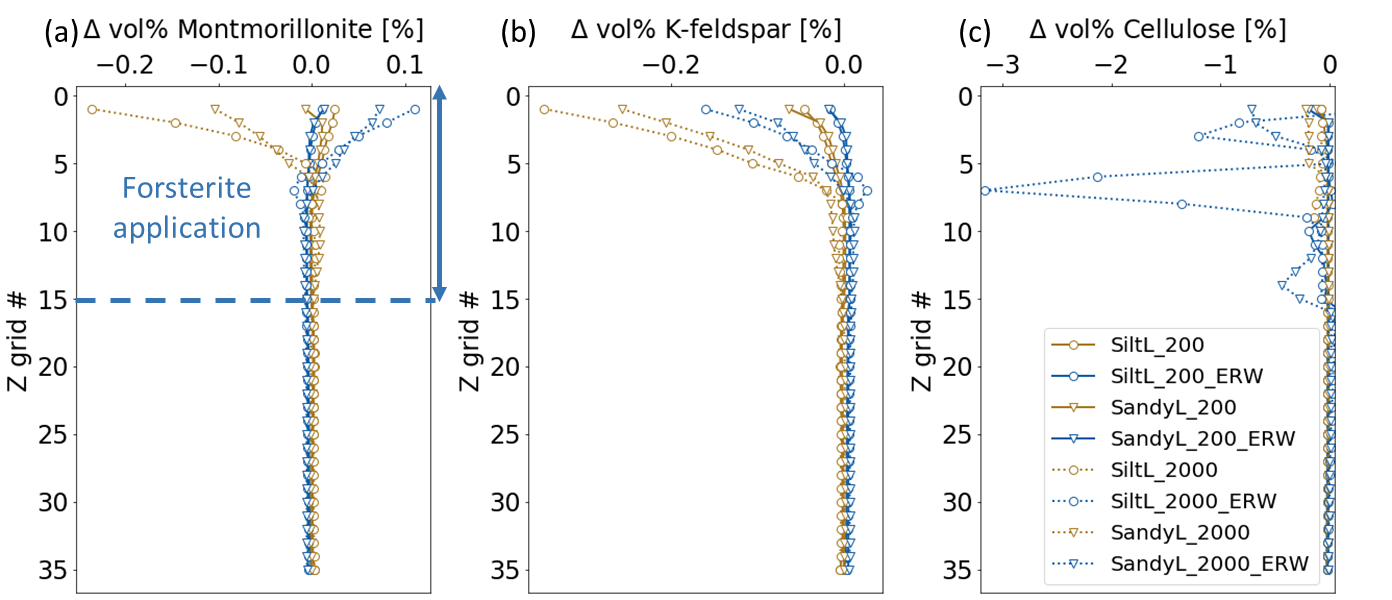


Figure S2. Vertical profiles at year five for an application of 15 cm forsterite with 10 m2/g surface area for both soil types at both infiltration rates. Changes in the volume fractions of (a) montmorillonite, (b) k-feldspar, and (c) cellulose.

## 4. Porosity Change

In our simulations it was assumed that the hydraulic properties do not evolve over time, i.e., porosity and permeability do not change. By tracking the mineral volume fractions, however, we can evaluate semi-quantitatively what porosity change might be expected. With an application depth of 15 and 50 cm in the high surface area cases, the porosity difference between the final and initial time steps ranges between -0.04 and 0.07, and -0.02 and 0.04, respectively. The increase in porosity is caused by net mineral removal by local dissolution, and the decrease in the porosity is caused by local precipitation (of e.g., magnesite and amorphous silica) and the fact that the molar volume of forsterite is smaller than the volume of the precipitation product, i.e., two moles of magnesite. With surficial application, porosity loss is dominant and, in some cases when water content is low and the fluid is highly supersaturated with respect to magnesite and silica, a complete porosity pugging might be expected. The reduction in porosity is likely to make the soil less-well drained and thus have a negative effect on weathering if the major CO_2_ source is atmosphere-based, and vice versa. However, for quantitative evaluations, updating the porosity and permeability during the simulations is necessary.

## References

1. Millington, R. J.; Quirk, J. P., Permeability of porous solids. *Transactions of the Faraday Society* **1961,** *57* (0), 1200-1207.

2. Lasaga, A. C., Fundamental approaches in describing mineral dissolution and precipitation rates. In *Chemical Weathering Rates of Silicate Minerals*, White, A. F.; Brantley, S. L., Eds. 1995; Vol. 31, pp 23-86.

3. Appelo, C. A. J. P. D., Geochemistry, groundwater and pollution. **2005**.

4. Waterhouse, H.; Arora, B.; Spycher, N. F.; Nico, P. S.; Ulrich, C.; Dahlke, H. E.; Horwath, W. R., Influence of Agricultural Managed Aquifer Recharge (AgMAR) and Stratigraphic Heterogeneities on Nitrate Reduction in the Deep Subsurface. *Water Resources Research* **2021,** *57* (5), e2020WR029148.

5. Palandri, J. L., *A compilation of rate parameters of water-mineral interaction kinetics for application to geochemical modeling [electronic resource] / by James L. Palandri and Yousif K. Kharaka ; prepared in cooperation with the National Energy Technology Laboratory, United States Department of Energy*. U.S. Dept. of the Interior, U.S. Geological Survey: Menlo Park, Calif, 2004.

6. Arora, B.; Spycher, N. F.; Steefel, C. I.; Molins, S.; Bill, M.; Conrad, M. E.; Dong, W.; Faybishenko, B.; Tokunaga, T. K.; Wan, J.; Williams, K. H.; Yabusaki, S. B., Influence of hydrological, biogeochemical and temperature transients on subsurface carbon fluxes in a flood plain environment. *Biogeochemistry* **2016,** *127* (2), 367-396.
